# *BRAF* and *NRAS* Mutations in Papillary Thyroid Carcinoma and Concordance in *BRAF* Mutations Between Primary and Corresponding Lymph Node Metastases

**DOI:** 10.1038/s41598-017-04948-3

**Published:** 2017-07-05

**Authors:** Najla Fakhruddin, Mark Jabbour, Michael Novy, Hani Tamim, Hisham Bahmad, Fadi Farhat, Ghazi Zaatari, Tarek Aridi, Gernot Kriegshauser, Christian Oberkanins, Rami Mahfouz

**Affiliations:** 1 Pathology and Laboratory Medicine Department of American University of Beirut Medical Center, Beirut, Lebanon; 2 Department of Pathology Hammoud Hospital University Medical Center, Saida, Lebanon; 3 ViennaLab Diagnostics GmbH, Vienna, Austria; 4 Department of Internal Medicine, American University of Beirut Medical Center, Beirut, Lebanon; 5 Department of Anatomy, Cell Biology and Physiological Sciences, Faculty of Medicine, American University of Beirut, Beirut, Lebanon; 6 Department of Oncology, Hammoud Hospital University Medical Center, Saida, Lebanon; 7 Faculty of Medicine, American University of Beirut, Beirut, Lebanon

## Abstract

Concordance between mutations in the primary papillary thyroid carcinoma (PTC) and the paired x lymph node metastasis may elucidate the potential role of molecular targeted therapy in advanced stages. *BRAF and NRAS* mutations in primary PTC (n = 253) with corresponding metastatic lymph node (n = 46) were analyzed utilizing StripAssays (ViennaLab Diagnostics). Statistical analysis was performed using (SPSS, Inc.), version 24.0 with a p-value of <0.05, and concordance via kappa agreement. *BRAF* mutation frequency in conventional PTC (cPTC): 56.8%, papillary thyroid microcarcinoma (PTMC): 36.5%, PTMC-FV: 2.7% and PTC-FV: 4.1%. *NRAS* mutation frequency in PTC-FV: 28.6%, PTMC: 28.6%, PTMC-FV: 23.8%, and cPTC: 19.0%. *BRAF* mutation correlation with older age in cPTC (42.6 versus 33.6) years (p < 0.001) was the only significant clinicopathologic parameter. *BRAF* mutations were concordant in the primary and its corresponding lymph node deposits in PTC with a kappa of 0.77 (p-value < 0.0001). *BRAF* mutations are predominant in cPTC and PTMC while *NRAS* mutations in PTC-FV. *BRAF* mutation is conserved in metastatic lymph node deposits, thus *BRAF* is an early mutational pathogenetic driver. Therefore, targeted therapy is potential in recurrent and advanced stage disease.

## Introduction

Papillary thyroid carcinoma (PTC) is the most common malignant thyroid cancer, accounting for 1.5% of all cancers in the United States^1^ and up to 6% in the Arab countries^2^. Around 50% of PTCs present with lymph node metastasis and approximately 5–7% show distant metastasis usually involving the lungs and bones^3^. Prognostic clinicopathologic factors in PTC, regarding recurrence and metastasis, include age, gender, tumor size, infiltrative growth pattern, multifocality, and extrathyroidal extension^4^. Recently, genetic aberrations have been postulated to be contributing factors to the clinical and behavioral metastatic risks of PTCs^5–7^. These include enzymes of the mitogen-activated protein kinase (MAPK) signaling pathway, specifically *BRAF* and *RAS* genes^8^. PTCs harboring the *BRAF* mutation are usually characterized by a T1799A point mutation in the v-Raf murine sarcoma viral oncogene homolog B1 (*BRAF*) resulting in a valine-to-glutamic acid switch at codon 600 (^*V600E*^)^9^.

Several studies reported an association of the BRAF mutational status with a number of PTC clinicopathologic parameters inclusive of recurrence and worse prognosis^10, 11^. For instance, Nikiforova *et al*. correlated between *BRAF* status on one hand and both advanced age (5^th^ decade) and extrathyroidal extension on the other hand^12^, while others reported no significant correlation^13, 14^. Furthermore, there is conflicting evidence with respect to *BRAF* mutational analysis on cytology smears as a guide for further surgical management. Alternative studies reported a prediction of lymph node status based on *BRAF* cytology^15, 16^, while Barabaro *et al*. identified no significant association^17^. Yet, there is increasing evidence that coexistence of this *BRAF* mutation with other promoter mutations, specifically *TERT* promoter mutations, might form a genetic background defining PTC with the worst clinicopathologic parameters and outcomes^18^. Specifically, the C228T *TERT* promoter mutation has been shown to be associated with the *BRAF* ^*V600E*^ mutation, which was prevalent in the aggressive types of thyroid cancer^19^.

In the era of targeted therapy, which is based on the understanding of tumor molecular biology, it is critical to determine the molecular profiles in both the primary and metastatic sites as well. The use of anti-*BRAF* therapy is currently under investigation in clinical trials for cases of advanced surgically unresectable and/or radioresistant thyroid cancer cases^20^. Therefore, we aimed in this study to determine the *BRAF* and *NRAS* molecular signature concordance rates between the four main different primary PTC subtypes and the corresponding paired metastatic lymph node deposits in order to elucidate the potential clinical implication of selective molecular targeted therapy in advanced stage PTC. Additionally, we sought to determine the frequencies and types of *BRAF* and *NRAS* mutations in a cohort of Lebanese patients and correlate between the findings and the various clinicopathologic features of individual PTC subtypes: conventional PTC (cPTC), papillary thyroid microcarcinoma (PTMC) defined as tumors measuring ≤1 cm in maximum diameter, follicular variant of PTMC (PTMC-FV) and the follicular variant of PTC (PTC-FV).

## Materials and Methods

### Patient Selection

All patients enrolled in this retrospective clinical study gave informed consents for both participation and publication of identifying information/images (when applicable). The study with all its experimental protocols was conducted under the Institutional Review Board (IRB) approvals of the American University of Beirut Medical Center (AUBMC) and Hammoud Hospital University Medical Center (HHUMC). All experiments were performed in accordance with relevant guidelines and regulations. Archived formalin fixed paraffin embedded (FFPE) tissues of 312 PTC patients were collected from the Departments of Pathology and Laboratory Medicine, AUBMC and HHUMC, Beirut, Lebanon, between the period of January 2001 and December 2011.

### Patients Tissue Sampling

**Figure 1:**
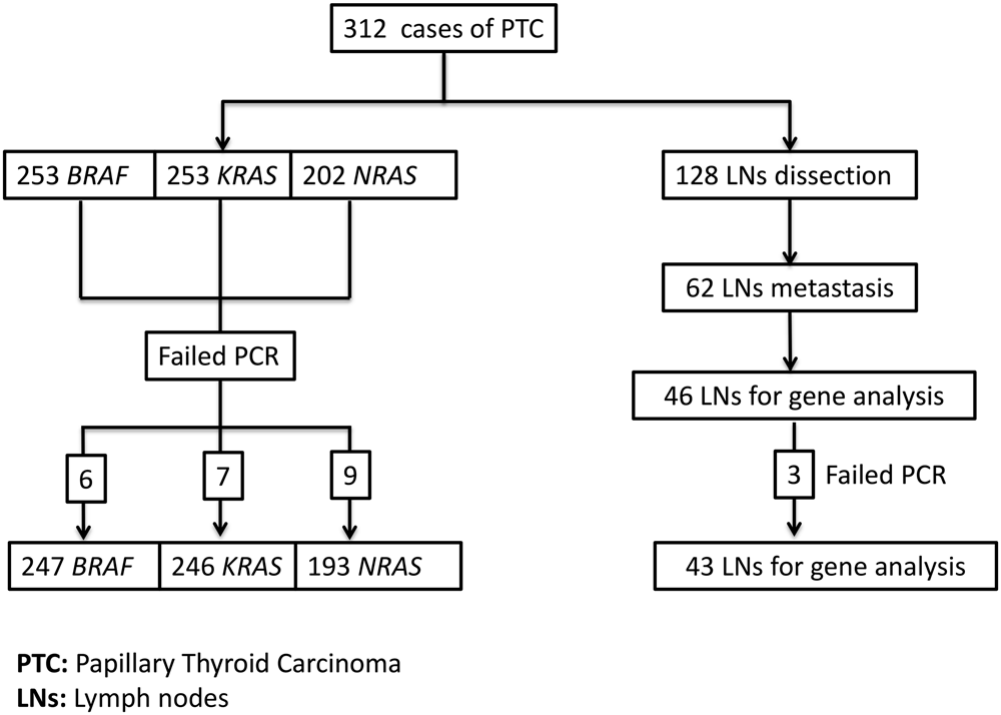
Stratification of the 312 cases of PTC included in our study.

Out of the 312 PTC cases, 253 PTC cases with available paraffin blocks and a minimal tumor size of 1 mm underwent mutational analysis. All the 253 PTC cases were analyzed for *BRAF* and *KRAS*, and only 202 with available extracted DNA underwent analysis for *NRAS* mutations (Fig. 1). As a negative control, 15 cases of multinodular goiter were used. Demographic (age and gender) and prognostic histopathologic features (tumor size, lymphovascular invasion, extrathyroidal invasion, focality, and lymph node metastasis) were evaluated and correlated with the molecular aberrations. Lymph node dissection was performed on128 cases of the 253, out of which 62 had metastatic lymph node deposits. Yet, only 46 cases with available paraffin blocks and a minimal tumor size of 1 mm underwent mutational analysis. Patients’ consents were waivered by the IRB because this is a retrospective study.

### DNA Extraction and Quantification

DNA was extracted from 253PTC cases utilizing the QIAamp FFPE DNA extraction kit (Qiagen, California, USA), and quantified via the Qubit fluorometer (Thermofisher Scientific, USA). The extracted DNA was stored at −20 °C until further use.

### BRAF, KRAS, and NRAS Analysis Using Reverse Hybridization

The *BRAF*, *KRAS*, and *NRAS* StripAssays (ViennaLab Diagnostic GmbH, Vienna, Austria) were utilized to detect different point mutations and deletions in the genes coding for *BRAF* and *NRAS*. The detection sensitivity for mutant alleles is 1%, performed according to the manufacturer’s instructions. Mutational analysis was performed by polymerase chain reaction (PCR) and reverse hybridization as follows: first, a multiplex PCR amplification using biotinylated oligonucleotide primers was performed for *BRAF, KRAS*, and *NRAS* gene sequence amplification; second, reverse hybridization of the amplification products was ensued via a test strip, which contains allele-specific oligonucleotide probes for mutations and controls immobilized on a parallel array; and finally, bound biotinylated sequences were visualized using streptavidin-alkaline phosphatase conjugate and enzymatic color development. Positive control samples included defined mutated cell line DNA or clones.

### Statistical Analysis

Data were entered into a Microsoft Excel datasheet, and then transferred to the Statistical Package for Social Science software (SPSS, Inc.), version 24.0, which was used for data management, cleaning, and analyses. Descriptive statistics was carried out and reported as number and percent for categorical variables, whereas the mean and standard deviation (±) for continuous ones. Association between mutation and demographic, clinical and pathological data was assessed using the Chi square test or Fisher’s exact test for categorical variables, and student’s independent t-test or Mann Whitney test for continuous ones. Moreover, to assess for the agreement between the primary tumor and its corresponding lymph node metastasis, kappa agreement was calculated and reported along with the p-value. Statistical significance was specified at 0.05 levels.

## Results

### Thyroid Cancer Patients’ Demographics

**Figure 2:**
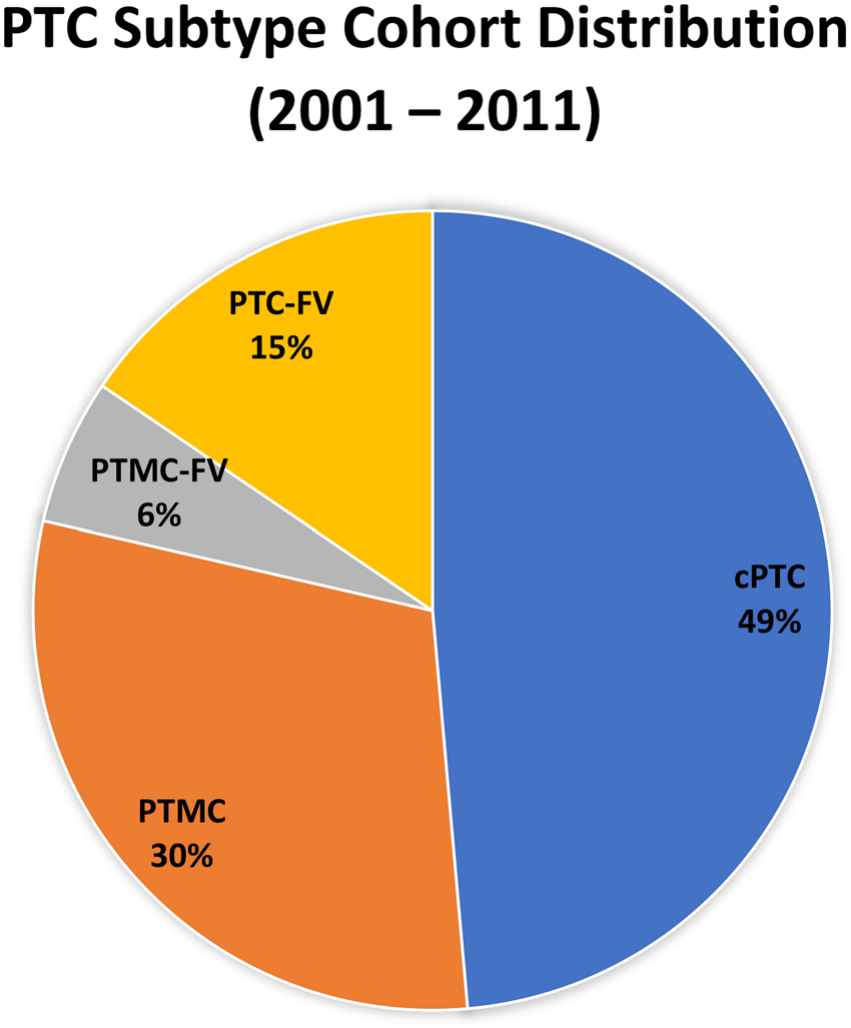
Pie graph showing the frequency of each PTC histopathologic subtype: 123 cases of cPTC (49%), 76 cases of PTMC (30%), 15 cases of PTMC-FV (6%), and 39 cases of PTC-FV (15%).

AUBMC thyroid carcinoma database from 2001 to 2011 revealed 385 thyroid cancers with PTC as the predominant type constituting 91.7% (321/385) of the cases. The overall female-to-male ratio of the PTC cases was 2.5:1. Approximately 26% of the patients were <30 years old, 56% were between 31–49 years old, and 18% were >50 years old. The frequency of each PTC histopathologic subtype was as follows: 123 cases of cPTC (49%), 76 cases of PTMC (30.0%), 15 cases of PTMC-FV (5.9%), and 39 cases of PTC-FV (15.5%) (Table 1, Fig. 2).

**Table 1:** Frequency of Primary *BRAF* and *NRAS* mutations in cPTC, PTMC, PTMC-FV and PTC-FV.

Variables	*BRAF* mutation n (*%*) n = 148	No *BRAF* mutation n (*%*) n = 99	P-value	*NRAS* mutation n (*%*) n = 21	No *NRAS* mutation n (*%*) n = 172	P-value
**PTC Subtype**			<0.0001*			<0.0001*
cPTC	84 (56.8)	36 (36.4)		4 (19.0)	97 (56.4)	
PTMC	54 (36.5)	21 (21.2)		6 (28.6)	49 (28.5)	
PTMC-FV	4 (2.7)	11 (11.1)		5 (23.8)	8 (4.7)	
PTC-FV	6 (4.1)	31 (31.3)		6 (28.6)	18 (10.5)	

*Significant difference.

### BRAF and NRAS Mutational Frequency

The frequency of *BRAF* and *NRAS* mutations varied among the different histopathologic subtypes of PTC. In cPTC and PTMC subtypes, *BRAF* mutations were predominant, while *NRAS* were less common. Conversely, in PTMC-FV and PTC-FV, *NRAS* mutations were more common than *BRAF* mutations with statistical significance of p < 0.0001. The *BRAF* ^*V600E*^ mutation subtype comprised 98.0% of the total *BRAF* mutated PTC cases followed by *BRAF* ^*V600M*^ identified in two cases: one cPTC case and a second cPTC case with a concomitant *BRAF* ^*V600E and V600M*^. The *NRAS* mutational subtypes included c.182 A > G (p.Q61R) c.181 C > A (p.Q61K), c.34 G > A (p.G12S) and c.38 G > A (p.G13D). Concomitant *BRAF* and *NRAS* mutations were detected in five PTC cases inclusive of one cPTC, two PTMC and two PTMC-FV cases. *KRAS* mutations were detected in only 4 out of 246 cases tested; therefore, no further statistical analysis was performed. No mutations were detected in all adenomatous goiter cases (n = 15).

### Clinicopathologic Correlation of Mutations with PTC Histopathologic Subtypes

The mutational status of *BRAF* and *NRAS* in the four PTC variants (cPTC, PTMC, PTMC-FV and PTC/FV) was compared to the clinicopathological parameters, including age, gender, tumor size, extracapsular extension, lymphovascular involvement, lymph node metastasis, and multifocality.

*BRAF* mutation in cPTC was significantly correlated to older age (*BRAF* mutated cPTC, mean age = 42.6 ± 14.5 years vs. wild-type cPTC, mean age = 33.6 ± 15.1 years, p = 0.005). A trend towards higher incidence of *BRAF mutation* was found in patients with higher tumor stage (p = 0.054). There was no significance association with respect to *BRAF* and *NRAS* mutations in the remaining cPTC clinicopathologic features (Table 2).

**Table 2:** Clinicopathological features of c-PTC cases with respect to *BRAF* and *NRAS* mutations.

Variables	*BRAF* mutation n (*%*) n=84		No *BRAF* mutation n (*%*) n=36		P-value	*NRAS* mutation n (*%*) n=4		No *NRAS* mutation n (*%*) n=97		P-value
**Age (years)**					0.005*					0.520
Mean ± SD	42.6±14.5		33.6±15.1			36.2±16.9		39.2±15.1		
**Gender**					0.120					0.306
Female	58	(69.0)	30	(83.3)		2	(50.0)	71	(73.2)	
Male	26	(31.0)	6	(16.7)		2	(50.0)	26	(26.8)	
**Stage**					0.054					0.612
I	62	(73.8)	31	(86.1)		3	(75.0)	77	(79.4)	
II	2	(2.4)	3	(8.3)		0	(0.0)	5	(5.2)	
III	14	(17.6)	1	(2.8)		1	(25.0)	10	(10.3)	
IV	4	(4.8)	0	(0.0)		0	(0.0)	4	(4.1)	
Not available	2	(2.4)	1	(2.8)		0	(0.0)	1	(1.0)	
**Focality**					0.338					0.659
Unifocal	54	(64.3)	24	(66.7)		2	(50.0)	61	(62.9)	
Multifocal	29	(34.5)	10	(27.8)		2	(50.0)	33	(34.0)	
Not available	1	(1.2)	2	(5.6)		0	(0.0)	3	(3.1)	0.592
**Size**					0.071					
≤3	72	(85.7)	26	(72.2)		3	(75.0)	78	(80.4)	
>3	11	(13.1)	7	(19.4)		1	(25.0)	15	(25.0)	
Not available	1	(1.2)	3	(8.3)		0	(0.0)	4	(4.1)	
**Extrathyroidal extension**					0.324					1.000
Present	34	(40.5)	13	(36.1)		2	(50.0)	40	(41.2)	
Absent	49	(58.3)	21	(58.3)		2	(50.0)	54	(55.7)	
Not available	1	(1.2)	2	(5.6)		0	(0.0)	3	(3.1)	
**Lymphovascular invasion**					0.196					0.252
Present	26	(31.0)	15	(44.4)		0	(0.0)	35	(36.1)	
Absent	35	(41.7)	16	(41.7)		3	(75.0)	35	(36.1)	
Not available	23	(27.4)	5	(13.9)		1	(25.0)	27	(27.8)	
**Lymphnodes status**					1.000					0.026
Positive	35	(41.7)	15	(41.7)		0	(0.0)	45	(34.0)	
Negative	24	(28.6)	11	(30.6)		1	(25.0)	33	(46.4)	
Not available	25	(29.8)	10	(27.8)		3	(75.0)	19	(19.6)	

*Significant difference.

**Figure 3:**
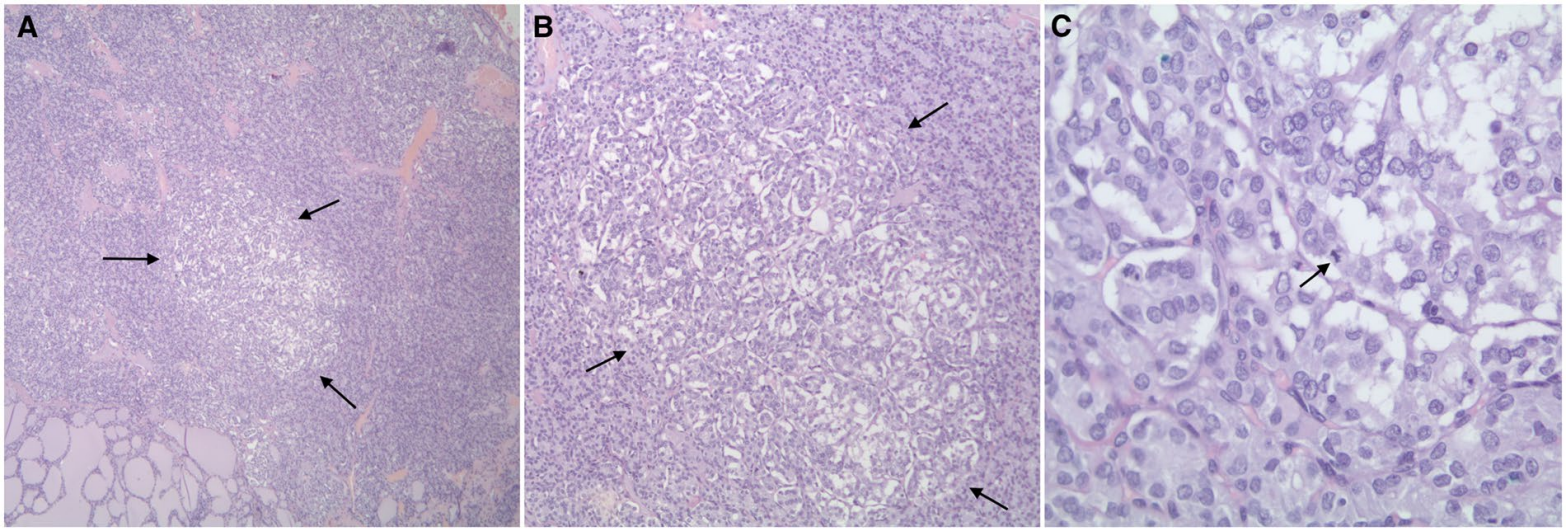
Histopathological examination of two PTC tissues. H&E staining (x100) shows A case of multifocal PTC-FV with a PTMC-FV focus that was positive for the *NRAS* mutation. (**A**) and (**B**) Note that the microscopic focus was unencapsulated (*arrows, 40x and 100x*). (**C**) Note the follicular architecture, irregular nuclei with clearing and grooves with a mitotic figure (*arrows, 400x*).

In PTMC and PTMC-FV, clinicopathologic parameters were not significantly correlated with neither *BRAF* nor *NRAS* mutations. However, there was a higher trend for *BRAF* mutation with multifocality in PTMC (Tables 3 and 4). Similarly, PTC-FV did not correlate with any clinicopathologic feature; however, we noticed that *BRAF* mutations were exclusive to tumors sizes smaller than or equal to 3 cm, absence of extrathyroidal extension, and absence of lymphovascular invasion, while *NRAS* mutations were exclusive to females and absence of extrathyroidal extension. (Fig. 3, Table 5).

**Table 3:** Clinicopathological features of PTMC cases with respect to *BRAF* and *NRAS* mutations.

Variables	*BRAF* mutation n (*%*) n = 54		No *BRAF* mutation n (*%*) n = 21		P-value	*NRAS* mutation n (*%*) n = 6		No *NRAS* mutation n (*%*) n = 49		P-value
**Age (years)**					0.42					0.88
Mean ± SD	46.6 ± 11.8		47.3 ± 14.9			45.0 ± 17.3		47.1 ± 11.6		
**Gender**					0.330					0.298
Female	43	(79.6)	19	(90.5)		4	(66.7)	41	(83.7)	
Male	11	(20.4)	2	(9.5)		2	(33.3)	8	(16.3)	
**Stage**					1.000					0.378
I	50	(92.6)	20	(95.2)		5	(83.3)	46	(93.9)	
II	0	(0.0)	0	(0.0)		0	(0.0)	0	(0.0)	
III	3	(5.6)	1	(4.8)		1	(16.7)	2	(4.1)	
IV	1	(1.9)	0	(0.0)		0	(0.0)	1	(2.0)	
Not available	0	(0.0)	0	(0.0)		0	(0.0)	0	(0.0)	
**Focality**					0.428					0.204
Unifocal	32	(59.3)	15	(71.4)		5	(83.3)	25	(51.0)	
Multifocal	22	(40.7)	6	(28.6)		1	(16.7)	24	(49.0)	
Not available	0	(0.0)	0	(0.0)		0	(0.0)	0	(0.0)	
**Extrathyroidal extension**					1.000					0.619
Present	11	(20.4)	4	(19.0)		2	(33.3)	11	(22.4)	
Absent	43	(79.6)	17	(81.0)		4	(66.7)	38	(77.6)	
Not available	0	(0.0)	0	(0.0)		0	(0.0)	0	(0.0)	
**Lymphovascular invasion**					0.850					0.339
Present	2	(3.7)	0	(0.0)		0	(0.0)	1	(2.0)	
Absent	46	(85.2)	18	(85.7)		4	(66.7)	41	(83.7)	
Not available	6	(11.1)	3	(14.3)		2	(33.3)	7	(14.3)	
**Lymphnodes status**					0.196					1.000
Positive	9	(16.7)	2	(9.5)		1	(16.7)	7	(14.3)	
Negative	14	(25.9)	2	(9.5)		1	(16.7)	13	(26.5)	
Not available	31	(57.4)	17	(81.0)		4	(66.7)	29	(59.2)	

*Significant difference.

**Table 4:** Clinicopathological features of PTMC-FV cases with respect to *BRAF* and *NRAS* mutations.

Variables	*BRAF* mutation n (*%*) n = 4		No *BRAF* mutation n (*%*) n = 11		P-value	*NRAS* mutation n (*%*) n = 5		No *NRAS* mutation n (*%*) n = 8		P-value
**Age (years)**					1.000					0.724
Mean ± SD	46.7 ± 14.7		47.3 ± 13.2			46.0 ± 14.2		44.4 ± 12.4		
**Gender**					1.000					0.385
Female	4	(100.0)	9	(81.8)		4	(80.0)	8	(100.0)	
Male	0	(0.0)	2	(18.2)		1	(20.0)	0	(0.0)	
**Stage**					0.476					1.000
I	3	(75.0)	10	(90.9)		4	(80.0)	7	(87.5)	
II	0	(0.0)	0	(0.0)		0	(0.0)	0	(0.0)	
III	1	(25.0)	1	(9.1)		1	(20.0)	1	(12.5)	
IV	0	(0.0)	0	(0.0)		0	(0.0)	0	(0.0)	
Not available	0	(0.0)	0	(0.0)		0	(0.0)	0	(0.0)	
**Focality**					0.604					0.565
Unifocal	3	(75.0)	6	(54.5)		4	(80.0)	4	(50.0)	
Multifocal	1	(25.0)	5	(45.5)		1	(20.0)	4	(50.0)	
Not available	0	(0.0)	0	(0.0)						
**Extrathyroidal extension**					0.476					1.000
Present	1	(25.0)	1	(9.1)		1	(20.0)	1	(12.5)	
Absent	3	(75.0)	10	(90.9)		4	(80.0)	7	(87.5)	
Not available	0	(0.0)	0	(0.0)		0	(0.0)	0	(0.0)	
**Lymphovascular invasion**					0.267					NA
Present	1	(25.0)	0	(0.0)			(0.0)		(0.0)	
Absent	3	(75.0)	11	(100.0)		5	(100.0)	8	(100.0)	
Not available	0	(0.0)	0	(0.0)		0	(0.0)	0	(0.0)	
**Lymphnodes status**					1.000					1.000
Positive	0	(0.0)	0	(0.0)		0	(0.0)	0	(0.0)	
Negative	1	(25.0)	2	(18.2)		1	(20.0)	2	(25.0)	
Not available	3	(75.0)	9	(81.8)		4	(80.0)	6	(75.0)	

*Significant difference.

**Table 5:** Clinicopathological features of PTC-FV cases with respect to *BRAF* and *NRAS* mutations.

Variables	*BRAF* mutation n (*%*) n = 6		No *BRAF* mutation n (*%*) n = 31		P-value	*NRAS* mutation n (*%*) n = 6		No *NRAS* mutation n (*%*) n = 18		P-value
**Age (years)**					0.952					0.974
Mean ± SD	40.8 ± 17.4		45.5 ± 12.9			41.5 ± 11.7		41.4 ± 12.9		
**Gender**					0.653					0.280
Female	4	(66.7)	23	(74.2)		6	(100.0)	13	(72.2)	
Male	2	(33.3)	8	(25.8)		0	(0.0)	5	(27.8)	
**Stage**					1.000					1.000
I	4	(66.7)	17	(54.8)		4	(66.7)	12	(66.7)	
II	1	(16.7)	8	(25.8)		1	(16.7)	3	(16.7)	
III	1	(16.7)	5	(16.1)		1	(16.7)	2	(11.1)	
IV	0	(0.0)	0	(0.0)		0	(0.0)	0	(0.0)	
Not available	0	(0.0)	1	(3.2)		0	(0.0)	1	(5.6)	
**Focality**					0.383					1.000
Unifocal	4	(66.7)	13	(41.9)		2	(33.3)	8	(44.4)	
Multifocal	2	(33.3)	18	(58.1)		4	(66.7)	10	(55.6)	
Not available	0	(0.0)	0	(0.0)		0	(0.0)	0	(0.0)	
**Size**					0.293					0.724
≤3	6	(100.0)	20	(64.5)		4	(66.7)	13	(72.2)	
>3	0	(0.0)	10	(32.3)		2	(33.3)	4	(22.2)	
Not available	0	(0.0)	1	(3.2)		0	(0.0)	1	(5.6)	
**Extrathyroidal extension**					0.571					0.546
Present	0	(0.0)	7	(22.6)		0	(0.0)	3	(16.7)	
Absent	6	(100.0)	24	(77.4)		6	(100.0)	15	(83.3)	
Not available	0	(0.0)	0	(0.0)		0	(0.0)	0	(0.0)	
**Lymphovascular invasion**					0.097					0.251
Present	0	(0.0)	7	(22.6)		2	(33.3)	1	(5.6)	
Absent	5	(83.3)	24	(77.4)		4	(66.7)	16	(88.9)	
Not available	1	(16.7)	0	(0.0)		0	(0.0)	1	(5.6)	
**Lymphnodes status**					0.307					1.000
Positive	1	(16.7)	1	(3.2)		0	(0.0)	2	(11.1)	
Negative	1	(16.7)	11	(35.5)		2	(33.3)	7	(38.9)	
Not available	4	(66.7)	19	(61.3)		4	(66.7)	9	(50.0)	

*Significant difference.

### BRAF Mutational concordance between primary PTC and paired lymph nodes metastasis

**Figure 4:**
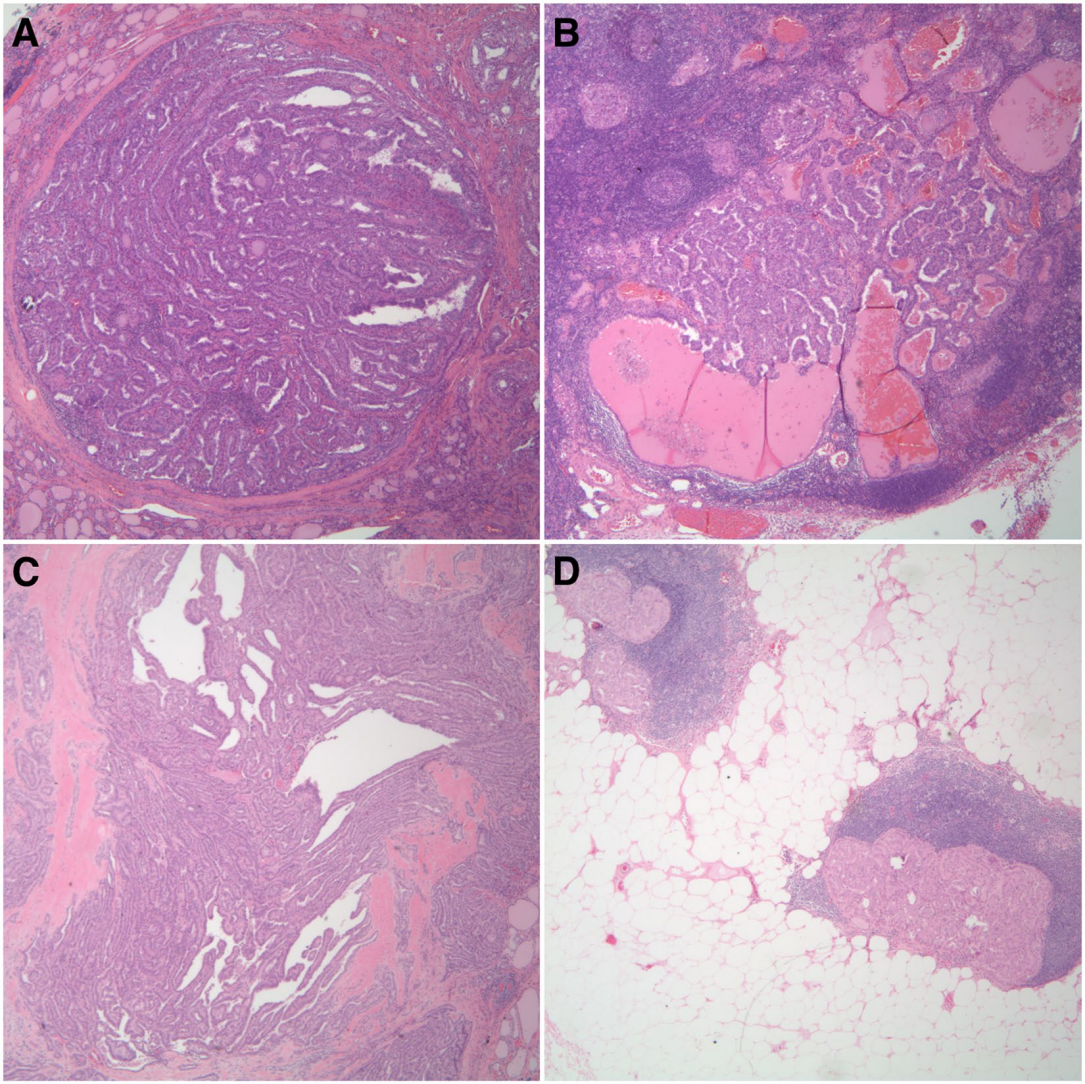
Histopathological examination of two PTC tissues. H&E staining (x100) shows (**A**) and (**B**) Representative case of mutant *BRAF* in primary cPTC (size = 3 cm and age = 28 years-old) and the corresponding paired lymph node metastasis (*40x*). (**C**) Primary PTMC (size = 0.7 cm and age = 33 years) with a mutant *BRAF* and (**D**) paired lymph node metastasis (*40x*).

*BRAF* mutations were concordant in the primary and its corresponding lymph node deposits in PTC with a kappa of 0.77 (p-value < 0.0001) (Fig. 4, Table 6). Agreement coefficients for mutational concordance between primary and paired lymph node deposits were not calculated for *NRAS* mutations due to the small number of *NRAS* mutated cases and their corresponding lymph node metastasis.

**Table 6:** Agreement in *BRAF* mutation in between primary PTC tumor and the corresponding metastatic lymph nodes.

		*Primary PTC*		Kappa (P-value)
		No *BRAF*mutation	*BRAF*Mutation	
**LN Metastasis**	No* BRAF *mutation	17 (94.4%)	4 (16.0%)	0.77 (<0.0001)
	*BRAF* Mutation	1 (5.6%)	21 (84.0%)	
	Total	18	25	

Concordance in *BRAF* mutation between primary PTC and the corresponding metastatic lymph nodes.

## Discussion

The current study evaluated the concordance rates of *BRAF* and *NRAS* mutations between primary PTC tumors and paired metastatic lymph node deposits of the four most common subtypes of PTC: cPTC, PTMC, PTMC-FV and PTC-FV. In addition, the mutational *BRAF* and *NRAS* statuses were correlated with the different clinicopathologic parameters.

*BRAF and RAS* mutations are the most common in PTC^21, 22^. In this series, we found that *BRAF* mutation incidence, approximated to be 60%, was closer to the higher edge of the worldwide reported range (36–69%), while *NRAS* was lower with approximately 11% vs. 30% reported in literature^23–26^. Comparably, we found that *BRAF* mutations were more prevalent than *NRAS* mutations in cPTC (56.8% vs. 50%) and PTMC (36.5% vs. 40%), whereas *NRAS* mutations showed a higher incidence than *BRAF* mutations in PTMC-FV (23.8%) and PTC-FV (28.6%). Interestingly, we identified a significantly elevated *NRAS* mutational frequency within PTMC (28.6%) similar to PTC-FV (28.6%); a finding higher than that reported by Schulten *et al*. (5.4%)^27^. Besides, among the 75 patients with PTMC evaluated in our cohort, 54 had *BRAF* mutation-positive (72% of PTMCs) while 21 had negative *BRAF* mutation (28% of PTMCs). Our results are in accordance with what has been reported in worldwide literature in this regards, where a study by Sun *et al*. showed that out of 86 PTMC cases, around 65% were positive for *BRAF* mutation^28^.

Clinicopathologic parameters’ correlation with *BRAF* and *NRAS* mutations is controversial among different studies^12–14^. In a cohort of 129 PTMCs tested for *BRAF* ^*V600E*^ mutation and their correlation with the clinicopathologic features of patients, results showed no significant differences in age, sex, tumor size, location, and multifocality between the *BRAF* ^*V600E*^ mutated and non-mutated microcarcinomas^9^. However, there was significantly higher prevalence of infiltrative tumor borders, tumor-associated stromal desmoplasia/fibrosis and/or sclerosis, classic nuclear features of PTC, and cystic change in mutated microcarcinomas^9^. Similarly, results from another study demonstrated significant association between *BRAF* mutation-positive tumors and the following features: infiltrative growth, stromal fibrosis, psammoma bodies, plump eosinophilic tumor cells, and classic fully developed nuclear features of PTC, but not other clinicopathological parameters^24^. In addition, *BRAF* mutational status has been correlated with recurrence of PTMCs, suggesting its importance in stratifying patients for surgical management^28, 29^. On the other hand, several papers concluded that *BRAF* positivity is not significantly associated with most clinicopathologic features redolent of aggressiveness, including tumor multicentricity, lymphovascular invasion, extranodal extension, central neck involvement, advanced stage (stage III or IV), and distant metastasis^30, 31^.

In our cases, the only significant clinicopathologic correlation found was between advanced age and *BRAF* mutation in cPTC (p < 0.005), a potential causal link between older age and an advanced stage disease presentation. While Rodolico *et al*. identified *BRAF* mutations in 41% of PTMCs and an association with a higher age (mean = 53 years) and lymph node metastasis^32^, we reported a frequency of 36.5% *BRAF* mutated cases in PTMCs but with no statistically significant correlation with the various clinicopathologic parameters. Yet, a trend towards higher incidence of *BRAF* mutation was found in patients with higher tumor stage (p = 0.054). That being said, the clinical benefit of selective molecular targeted therapy in aggressive and advanced stage PTMC is still questionable^33^.

PTC-FV, which was initially described by Lindsay *et al*.^34^ and categorized by Chem and Rosai due to the morphologic and biological overlap with PTC^35^, represents a unique molecular subgroup of PTC cases. At the molecular level, and in contrast to cPTC and PTMC, PTC-FV exhibits a *RAS* family mutation. The *Cancer Genome Atlas* clustered PTC into two main morphologically and molecularly distinct groups, namely *BRAF* driven and *RAS* mutated tumors^36^. Nikiforov *et al*. recommends that the encapsulated variant of PTC-FV is best classified as “noninvasive follicular thyroid neoplasm with papillary-like nuclear features” (NIFTP) due to the low risk malignant behavior. Only cases with the infiltrative pattern retain the PTC-FV term^37^. One case of PTC-FV harbored lymph node metastasis and was negative for the *BRAF* or *NRAS* mutation, while none of the PTMC-FV cases exhibited lymph node metastasis. The literature on lymph node metastasis in PTC-FV varies greatly among different studies and ranges between14% and 94%^38^.

Locoregional lymph node metastasis in PTC may be found in up to 46.8%^39^. In high-risk patients, characterized by older age, tumor size >3 cm, and extracapsular extension, the number and size of lymph node metastasis affects prognosis and survival. Locoregional recurrence, with a follow-up of three decades, can reach up to 30%^40–42^. The current study showed a highly significant concordance rate of 84% for *BRAF* mutation in primary PTC and corresponding paired lymph node metastasis. Similarly, Walts *et al*. and Vasco *et al*. reported concordance rates of 95.2% and 81% respectively for primary PTCs and the corresponding paired metastatic lymph node deposits^43, 44^. This implies that *BRAF* mutation is conserved in both the primary and paired metastatic lymph nodes, thus supporting the hypothesis of a driver mutational role in the pathogenesis of PTC, particularly cPTC and PTMC, a finding reinforced by the genomic analysis of PTC via the *Cancer Genome Atlas Research Network* ^36^. Therefore, does *BRAF* testing predict central lymph node metastasis and an aggressive PTC phenotype? Actually, the positive predictive value and negative predictive values of *BRAF* mutational testing in PTC as a marker of central lymph node metastasis were estimated to be 47% and 91%, respectively^45^. Hence, the utility of *BRAF* as a prognostic marker may be confined to the cPTC subtype^46^.

Argumentatively, there is a potential role of selective molecular targeted therapy in recurrent and advanced metastatic PTC cases that are surgically unresectable and radioresistant. Phase II clinical trials utilizing Selumetinib, a tyrosine kinase inhibitor targeting *BRAF* mutations in PTC, were conducted without any significant survival benefit^47^. Currently, a study by Dadu *et al*. involving treatment of advanced cPTC stage disease exhibited a 47% partial response and a 53% stable disease over a minimal 6-month period^48^. The *BRAF* status of the paired lymph node deposits was not determined in the study by Dadu *et al*. An interesting prospective study may identify responders versus non-responders with respect to metastatic lymph node *BRAF* status. In our study, *NRAS* mutations within metastatic lymph nodes were detected only in cPTC and PTMC, but the numbers are too low to conclude a significant concordance rate in either.

This study carries a number of limitations that relate to the relatively small number of cases evaluated, especially PTMC-FV and PTC-FV cases, and accordingly data may not apply to the different subtypes of PTC. Besides, the study is also limited by being retrospective in nature.

## Conclusion

In conclusion, *BRAF* mutation is conserved in the primary and paired metastatic lymph node deposits in cPTC and PTMC. Testing for the *BRAF* mutation within lymph nodes is recommended in order to identify responders to the selective tyrosine kinase inhibitors in advanced stage cPTC. The high prevalence of *BRAF* and *NRAS* in PTMC and PTMC-FV with the absent significant clinicopathologic correlation undermines the role of *BRAF* testing in such a predominantly curable malignant thyroid disease. Finally, *NRAS* and *BRAF* testing in PTC-FV comprise a potentially diagnostically reassuring result. Further prospective studies are required to assess *BRAF* status within primary and paired lymph nodes for patients treated with selective targeted therapy in advanced stage cPTC.
